# *Cis*-2-dodecenoic acid quorum sensing system modulates *N*-acyl homoserine lactone production through RpfR and cyclic di-GMP turnover in *Burkholderia cenocepacia*

**DOI:** 10.1186/1471-2180-13-148

**Published:** 2013-07-01

**Authors:** Yinyue Deng, Amy Lim, Jing Wang, Tielin Zhou, Shaohua Chen, Jasmine Lee, Yi-Hu Dong, Lian-Hui Zhang

**Affiliations:** 1 Institute of Molecular and Cell Biology, 61 Biopolis Drive, Proteos 138673, Singapore

## Abstract

**Background:**

*Burkholderia cenocepacia* employs both N-Acyl homoserine lactone (AHL) and *cis*-2-dodecenoic acid (BDSF) quorum sensing (QS) systems in regulation of bacterial virulence. It was shown recently that disruption of BDSF synthase RpfF_Bc_ caused a reduction of AHL signal production in *B. cenocepacia*. However, how BDSF system influences AHL system is still not clear.

**Results:**

We show here that BDSF system controls AHL system through a novel signaling mechanism. Null mutation of either the BDSF synthase, RpfF_Bc_, or the BDSF receptor, RpfR, caused a substantial down-regulation of AHL signal production in *B. cenocepacia* strain H111. Genetic and biochemical analyses showed that BDSF system controls AHL signal production through the transcriptional regulation of the AHL synthase gene *cepI* by modulating the intracellular level of second messenger cyclic di-GMP (c-di-GMP). Furthermore, we show that BDSF and AHL systems have a cumulative role in the regulation of various biological functions, including swarming motility, biofilm formation and virulence factor production, and exogenous addition of either BDSF or AHL signal molecules could only partially rescue the changed phenotypes of the double deletion mutant defective in BDSF and AHL signal production.

**Conclusions:**

These results, together with our previous findings, thus depict a molecular mechanism with which BDSF regulates AHL signal production and bacterial virulence through modulating the phosphodiesterase activity of its receptor RpfR to influence the intracellular level of c-di-GMP.

## Background

Quorum sensing (QS) is widely employed by bacterial pathogens to coordinate bacterial group behavior and regulate biological functions such as biofilm formation, motility, virulence, plasmid transfer, and antibiotic production [1,2]. This regulation mechanism depends on the production and perception of diffusible signal molecules in a cell-density dependent manner [2-4]. At low cell density, bacterial cells produce a basal level of QS signals, which are diffused or transported into extracellular environments. When the cell density reaches a critical concentration, the accumulated signals initiate a set of biological activities in a coordinated fashion. Several types of QS systems have been identified including the most-characterized acylhomoserine lactone (AHL) dependent QS system and the relatively newly identified diffusible signal factor (DSF) dependent QS system [3,5]. The AHL- and DSF-QS systems are mainly conserved in different Gram-negative bacteria pathogens.

While most bacterial pathogens employ either AHL- or DSF-dependent QS systems in regulation of virulence and biofilm formation [3,6], the members of the *Burkholderia cepacia* complex were found to produce both AHL- and DSF-type QS signals [7-9]. In *B. cenocepacia*, which is an opportunistic pathogen in cystic fibrosis or immunocompromised patients, the AHL-type QS system comprises the AHL synthase CepI, which was shown to catalyze the synthesis of *N*-octanoyl homoserine lactone (C8HSL, also known as OHL) as a major AHL signal [10,11], and the AHL receptor CepR. The receptor CepR forms a complex with AHL signals to activate or repress a set of target genes, and thus control a range of biological functions, including virulence, swarming motility and biofilm formation [8,9].

In addition to the AHL-dependent QS system, a DSF-dependent system has recently been identified in *B. cenocepacia*[12-15]. The QS signal synthase, RpfF_Bc_, catalyzes the production of BDSF signal (*cis*-2-dodecenoic acid), which is an analogue of the QS signal DSF (*cis*-11-methyl-2-dodecenoic acid), originally identified in the plant bacterial pathogen *Xanthomonas campestris* pv. *campestris*[16]. Our recent study showed that BDSF acts by interacting with its receptor RpfR, which is a modular protein with PAS-GGDEF-EAL domains [14]. Perception of BDSF by RpfR sharply enhances its c-di-GMP phosphodiesterase activity and consequently causes a reduction in the intracellular level of the second messenger cyclic di-GMP (c-di-GMP) in *B. cenocepacia*, which consequently affects a range of biological activities, including swarming motility, biofilm formation and virulence [14].

It has become clear that both AHL and BDSF systems control similar biological functions. Recently, it was reported that there is a direct relationship between the two QS systems as inactivation of BDSF synthase reduces the production of AHL signals in *B. cenocepacia*[17,18]. However, how BDSF system affects AHL system remains obscure. In this study, by generating and analyzing single- and double-deletion mutants defective in QS signal production, we showed that BDSF signaling system plays a dominant role in the regulation of AHL QS system and various biological activities in *B. cenocepacia*. In addition, we have investigated the molecular mechanisms with which BDSF signaling system influencing AHL signal production and unveiled the involvement of the second messenger c-di-GMP. Furthermore, we have determined the relationships of these two QS systems in the cell-cell communication signaling cascade and their impacts on bacterial physiology and virulence.

## Results

### BDSF system positively regulates AHL signal production

**Figure 1:**
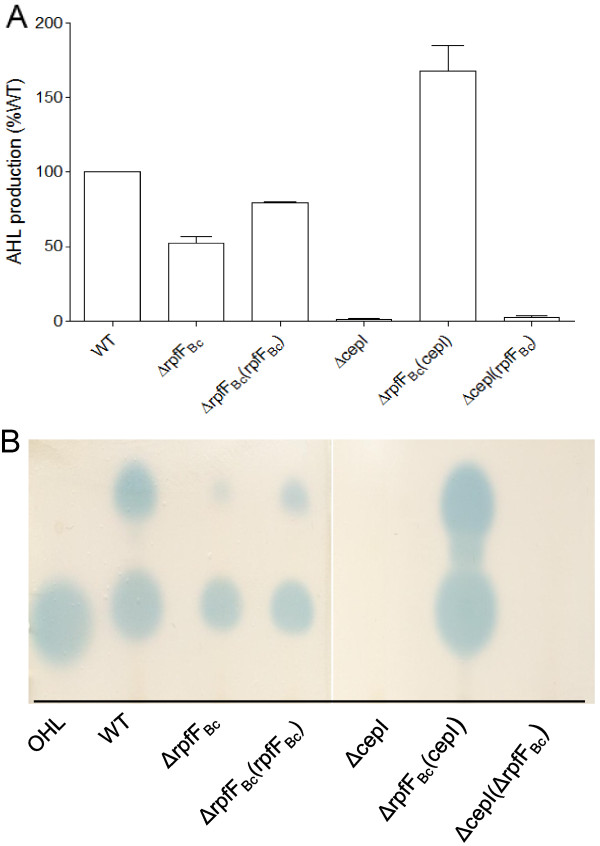
**Influence of the BDSF system on AHL signal production. (A)** AHL signal production was quantified with the aid of AHL reporter strain CF11 to test the β-galactosidase activity. **(B)** TLC assay of AHL signal production. For convenient comparison, the AHL signal production of wild-type strain was defined as 100% and used to normalize the AHL signal production of other strains. The data presented are the means of three replicates and error bars represents the standard deviation.

To further confirm whether the AHL and BDSF systems are functionally related, we determined the AHL and BDSF signal production levels in corresponding mutants. Consistently, we found deletion of either the AHL synthase gene *cepI* or the AHL receptor gene *cepR* had no effect on BDSF production (data no shown). However, we found that disruption of the BDSF synthase gene *rpfF*_*Bc*_ in *B. cenocepacia* H111 caused a significant reduction of the total AHL signal level with the aid of AHL reporter strain (Figure 1A). BDSF production was restored by *in trans* expression of the wild type *rpfF*_*Bc*_ (Figure 1A)*,* confirming the role of BDSF system in regulation of AHL biosynthesis. In contrast, *in trans* expression of *rpfF*_*Bc*_ in the *cepI* deletion mutant displayed no effect, suggesting that BDSF probably functions through modulation of CepI expression level or enzyme activity. Furthermore, we used the TLC method to analyze the different AHL signals produced by these strains. Results showed that deletion of *rpfF*_*Bc*_ affected the production of both HHL and OHL signals in *B. cenocepacia* H111 (Figure 1B).

### BDSF system positively controls *cepI* expression at transcriptional level

**Figure 2:**
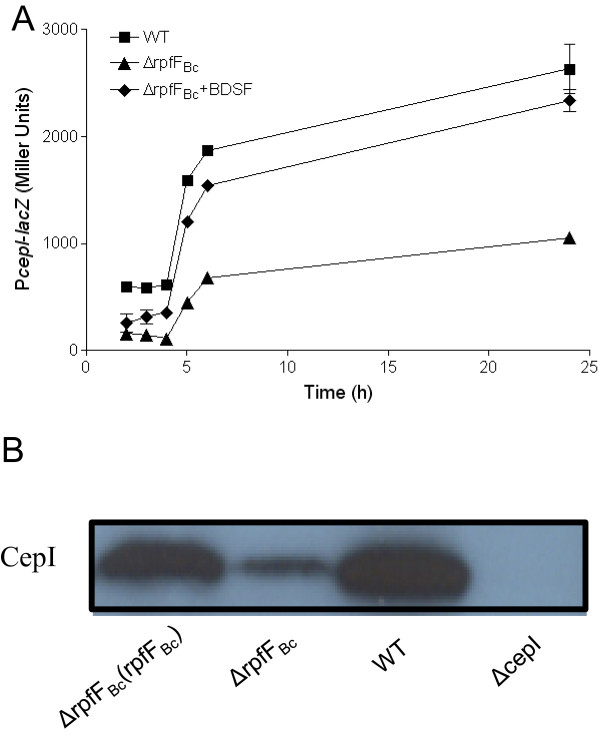
**Effect of RpfF**_**Bc **_**on AHL synthase gene *cepI *expression. (A)** The β-galactosidase activity of a *cepI*-*lacZ* transcriptional fusion in H111 wild-type (■), ∆rpfF_Bc_ (▲) and ∆rpfF_Bc_ supplemented with BDSF signal (◆). **(B)** Western blotting assay of CepI protein level. The data presented are the means of three replicates and error bars represents the standard deviation.

To further study the regulation mechanism of the BDSF system on AHL signal production, we constructed the *cepI* reporter system in *B. cenocepacia* H111 strains to test whether BDSF system controls *cepI* expression at transcriptional level. In agreement with the above results, deletion of *rpfF*_*Bc*_ resulted in a reduced expression of *cepI* at various growth stages (Figure 2A). Exogenous addition of BDSF rescued the *cepI* expression in ΔrpfF_Bc_ close to the wild-type level (Figure 2A). In agreement with the above results, western blotting analysis showed that null mutation of RpfF_Bc_ substantially decreased the CepI protein level (Figure 2B). These data established a positive regulatory role of the BDSF-dependent QS system in modulation of the *cepI* transcriptional expression.

**Figure 3:**
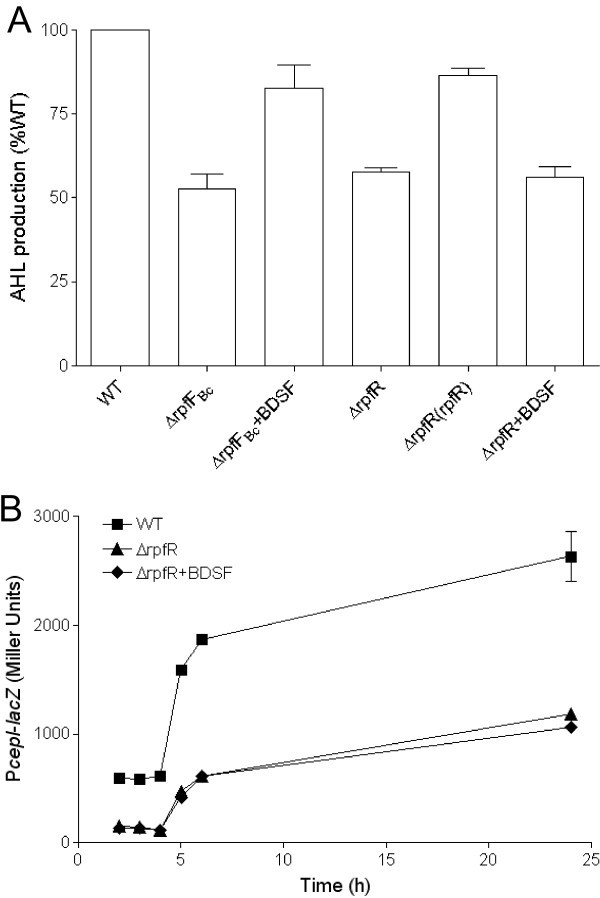
**Effect of RpfR on AHL system. (A)** AHL signal production was quantified with the aid of AHL reporter strain CF11 to test the β-galactosidase activity. **(B)** The β-galactosidase activity of a *cepI*-*lacZ* transcriptional fusion in H111 wild-type (■), ∆rpfR (▲) and ∆rpfR supplemented with BDSF signal (◆). For convenient comparison, the AHL signal production of wild-type strain was defined as 100% and used to normalize the AHL signal production of other strains. The data presented are the means of three replicates and error bars represents the standard deviation.

BDSF system controls AHL signal production through its receptor RpfR Previous studies showed that two BDSF sensors, BCAM0227 and RpfR (BCAM0580), are involved in the BDSF-mediated QS. Among them, BCAM0227, which was originally characterized in *B. cenocepacia* strain J2315, controls only a subset of the BDSF-regulated phenotypes and target genes [19], whereas RpfR was shown to be a major receptor of BDSF as null mutation of RpfR results in similar mutant phenotypes as the BDSF-minus mutants [14]. These results suggest that two BDSF signaling pathways may be operating in *B. cenocepacia,* which motivated us to investigate which BDSF signaling pathway plays a role in regulation of the *cepI* expression. Significantly, deletion of the BDSF receptor gene *rpfR* caused a similar reduction in AHL signal production as the deletion mutant of *rpfF*_*Bc*_ that encodes a BDSF synthase (Figure 3A). Analysis of the *cepI* expression profile using its promoter fused with the *lacZ* reporter gene showed that RpfR controlled the *cepI* expression at the transcriptional level (Figure 3B). Importantly, in contrast to the deletion mutant of *rpfF*_*Bc*_, which could be rescued by addition of BDSF (Figure 2A), addition of BDSF to the *rpfR* mutant had no effect on the *cepI* expression (Figure 3B). The data are consistent with the idea that BDSF modulates AHL signal production through its cognate receptor RpfR. Agreeable with our recent finding that BCAM0227 has a negligible role in BDSF signaling [14], deletion of this gene did not reveal any effect on *cepI* expression in *B. cenocepacia* H111 (Additional file 1: Figure S1).

### BDSF system controls AHL signal production and biological functions through regulation of intracellular c-di-GMP level

**Figure 4:**
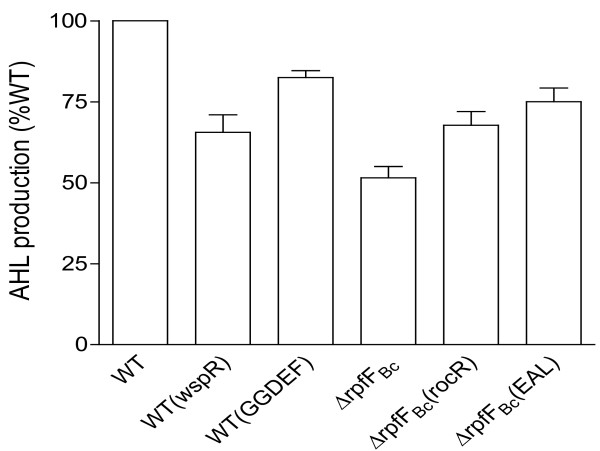
**Effect of intracellular c-di-GMP level on AHL signal production. ***In trans* expression of the c-di-GMP synthases, WspR from *P. aeruginosa* or the GGDEF domain of RpfR, in wild type H111 led to decreased AHL signal production; while overexpression of the c-di-GMP phosphodiesterases, RocR from *P. aeruginosa* or the EAL domain of RpfR resulted in increased AHL signal biosynthesis in BDSF-minus mutant ∆rpfF_Bc_. Quantification of AHL signal production was performed with the aid of AHL reporter strain CF11. For convenient comparison, the AHL signal production of wild-type strain was defined as 100% and used to normalize the AHL signal production of other strains. The data presented are the means of three replicates and error bars represents the standard deviation.

RpfR is a modular protein with PAS-GGDEF-EAL domains. Among these domains, PAS is the domain interacting with BDSF, and GGDEF and EAL domains are associated with c-di-GMP metabolism [14]. To investigate the regulation of RpfR in AHL signal production in detail, critical catalytic residues of either the GGDEF or the EAL domain were mutagenized. *In trans* expression of RpfR harboring a mutation in the GGDEF motif (changed to GGAAF) complemented the AHL signal production defects of the rpfR mutant (Additional file 2: Figure S2). In contrast, mutation of the EAL motif (changed to AAL) failed to complement the AHL signal production of the rpfR mutant (Additional file 2: Figure S2), To further confirm the change of intracellular c-di-GMP level could affect AHL signal production, we expressed *in trans* the *wspR* gene from *Pseudomonas aeruginosa*, which encodes a well-characterized c-di-GMP synthase [20], and the DNA sequences encoding the GGDEF domain of RpfR in *B. cenocepacia* wild-type strain H111. Bioassay results showed that increasing intracellular level of c-di-GMP by expressing either the c-di-GMP synthase WspR or the GGDEF domain of RpfR in *B. cenocepacia* wild-type strain H111 caused a reduction of AHL signal production by about 34% and 18%, respectively, compared with the wild type control containing empty vector only (Figure 4). We then *in trans* expressed the *rocR* gene from *P. aeruginosa* encoding a known c-di-GMP phosphodiesterase [21], and the DNA fragment encoding the EAL domain of RpfR in the BDSF-minus mutant ΔrpfF_Bc_, separately. The results showed that decreasing the intracellular c-di-GMP level by expression of c-di-GMP degradation proteins RocR and the EAL of RpfR increased AHL signal production by about 29% and 46%, respectively, compared with the parental strain ΔrpfF_Bc_ (Figure 4). We have shown previously that *in trans* expression of the c-di-GMP synthase GGDEF domain of RpfR diminished the swarming motility, biofilm formation, and protease activity of △rpfF_Bc_, whereas *in tans* expression of RocR, a c-di-GMP phosphodiesterase, significantly increased the motility, biofilm formation and protease production of ∆rpfF_Bc_[14]. Similarly, we found that *in trans* expression of the c-di-GMP synthase WspR diminished the swarming motility (Additional file 3: Figure S3A), biofilm formation (Additional file 3: Figure S3B), and protease activity (Additional file 3: Figure S3C) of ∆rpfF_Bc_ to the level of double deletion mutant ∆rpfF_Bc_∆cepI, whereas *in tans* expression of RocR, a c-di-GMP phosphodiesterase, significantly increased the motility, biofilm formation and protease production of ∆rpfF_Bc_ (Additional file 3: Figure S3A-C). Taken together, these results demonstrated that BDSF system controls AHL signal production and influences the bacterial physiology *via* modulation of the intracellular c-di-GMP level in *B. cenocepacia* H111.

### The cumulative effect BDSF and AHL systems on regulation of bacterial motility, biofilm formation and protease activity

**Figure 5:**
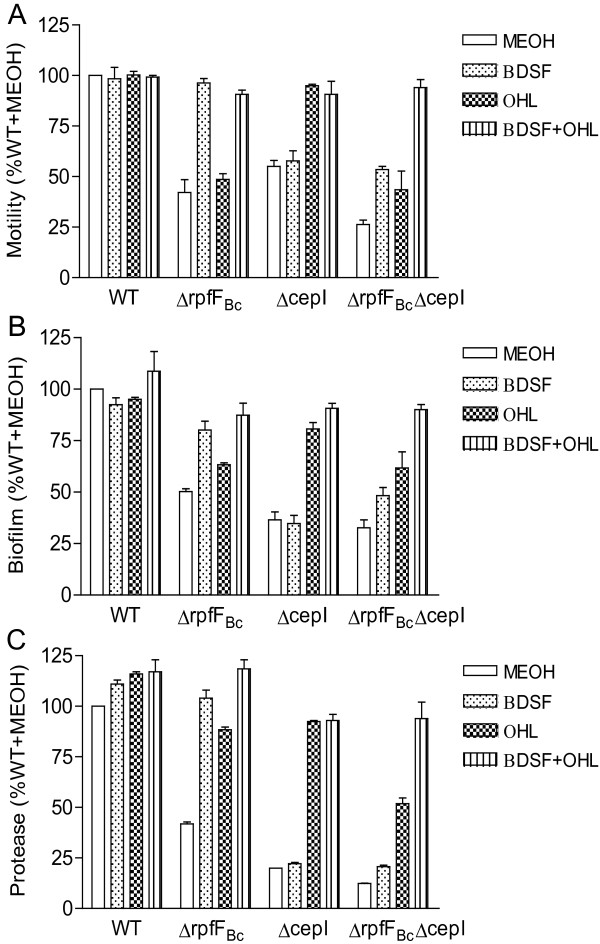
**Cumulative effect of the BDSF and AHL systems in regulation of bacterial motility (A), biofilm formation (B), and protease production (C).** For convenient comparison, these activity values of wild-type strain were defined as 100% and used to normalize the activities of other strains. The data presented are the means of three replicates and error bars represents the standard deviation.

To understand how AHL and BDSF systems function in regulation of bacterial biological activities, we compared the phenotype changes of the wild type strain H111, single deletion mutants of *rpfF*_*Bc*_ and *cepI*, and the double deletion mutant of *rpfF*_*Bc*_ and *cepI*, in the presence and absence of BDSF signal and OHL signal, respectively. As shown in Figure 5A-C, exogenous addition of 5 μM OHL or BDSF showed no evident effect on the phenotypes of wild type strain, suggesting that both signals were produced by H111 at “saturated” levels under the experimental conditions used in this study. As expected, addition of the same amount of OHL or BDSF to the corresponding AHL-minus and BDSF-minus mutants restored the mutants phenotypes including swarming motility (Figure 5A), biofilm formation (Figure 5B), and protease activity (Figure 5C). It was noticed that exogenous addition of BDSF to the AHL-minus mutant ΔcepI failed to rescue the changed phenotypes (Figure 5A-C). This could be explained that the mutant ΔcepI produced a similar “saturated” level of BDSF as the wild type, thus extra addition of BDSF had no effect in phenotype restoration. Interestingly, two different responses were noticed when OHL was added to the BDSF-minus mutant ΔrpfF_Bc_. While exogenous addition of the OHL signal could partially or even largely restore the biofilm formation and protease activity of this BDSF-minus mutant (Figure 5B, 5C), exogenous addition of OHL had no effect on the swarming motility of ΔrpfF_Bc_ (Figure 5A). One plausible hypothesis is that regulation of bacterial motility requires only a low level of AHL signals and the BDSF-minus mutant could still produce sufficient amount of AHL signal molecules above the “threshold” level for full activation of the AHL-dependent motility, whereas in the cases of biofilm formation and protease activity deletion of *rpfF*_*Bc*_ dropped the AHL level below the “threshold” concentration for full activation so that extra AHL addition could partially rescue the changed phenotypes. Consisting with the involvement of both BDSF and AHL systems in regulation of bacterial physiology, a cumulative effect on motility, biofilm formation and protease activity became evident when both *rpfF*_*Bc*_ and *cepI* were knocked out (Figure 5A-C). Significantly, only addition of both BDSF and OHL together could fully rescue the changed phenotypes of the double deletion mutant ΔrpfF_Bc_ΔcepI (Figure 5A-C).

### The impact of BDSF and AHL signaling systems on *B. cenocepacia* H111 pathogenicity

**Figure 6:**
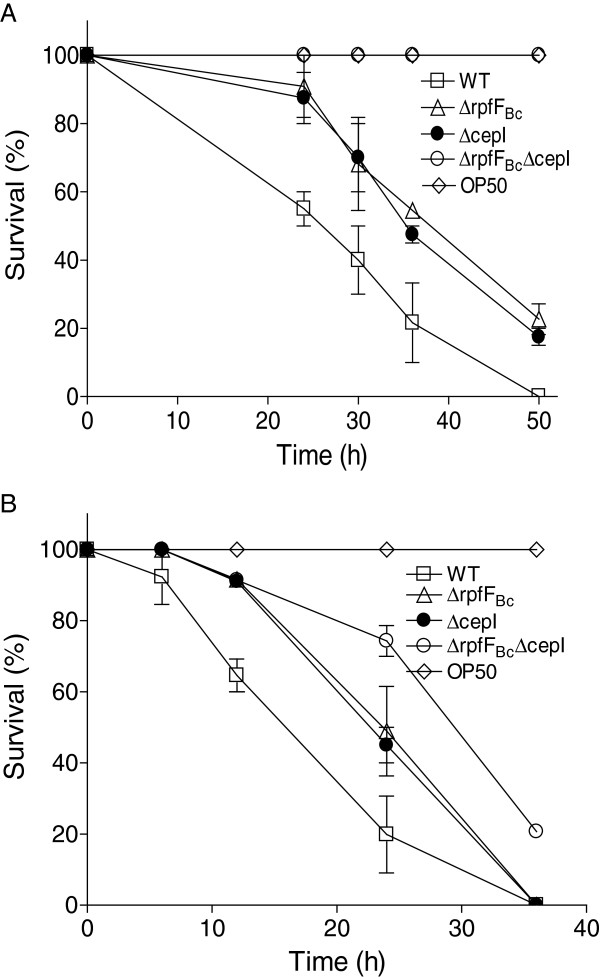
**Influence of RpfF**_**Bc **_**and CepI on the virulence of *B. cenocepacia *against *C. elegans. *(A)** Mutants ∆rpfF_Bc_ (∆), ∆cepI (●) and ∆rpfF_Bc_∆cepI (○) showed the reduced virulence compared with their parental wild-type strain H111 (□) in slow killing (A) and fast killing **(B)** assays. OP50 was used as the mock control. The data presented are the mean of triplicate experiments and the error bars represents the standard deviations.

The impact of BDSF and AHL systems on *B. cenocepacia* virulence was evaluated by using *C. elegans* infection models. Agreeable with the previous reports [14,22], deletion of either *rpfF*_*Bc*_ or *cepI* led to an reduction of virulence in both slow killing and fast killing assays of *C. elegans* (Figure 6A, 6B)*.* Remarkably, deletion of both *rpfF*_*Bc*_ and *cepI* completely or almost completely abolished the bacterial virulence against *C. elegans* (Figure 6A, 6B).

## Discussion

Many bacterial pathogens contain either AHL- or DSF-type QS systems in coordination of bacterial physiology. The human opportunistic pathogen *B. cenocepacia* is one of the exceptions which contain both BDSF and AHL signaling mechanisms [7,12,13,15,19,23]. In this study, we have investigated the relationship of the two QS systems in signaling modulation of bacterial physiology and virulence. Although the recently published results believe that the BDSF and AHL systems control overlap set and specific genes [17,18], we found that the two QS systems exert cumulative effect on bacterial motility, biofilm formation and virulence factor production (Figure 5A-C). In addition, we showed that BDSF regulates AHL signal production by influencing the c-di-GMP phosphodiesterase activity of its receptor RpfR. Given that both QS systems are widely conserved in the members of *B. cepacia* complex [7,10], it would be of great interest to investigate whether the similar cross-talking mechanisms of the AHL and BDSF systems are conserved in other members of the *Burkholderia* species.

The intracellular signal c-di-GMP is a widely conserved second messenger, which is known to be involved in the regulation of a range of biological activities, including bacterial motility, biofilm formation and virulence factor production [10,24,25]. The research progress over the last few years shows that c-di-GMP commonly controls various biological functions through interacting with different receptor or effector proteins, such as PilZ, FleQ, VpsT, LapD, FimX, PelD, and Clp [26-32]. Interestingly, different from this paradigm, the findings from this study have unveiled a new mechanism with which c-di-GMP could influence bacterial physiology. We showed that null mutation of RpfR, which is an one-component BDSF sensor/response regulator containing a BDSF-binding domain and the GGDEF-EAL domains associated with c-di-GMP metabolism [14], resulted in a similar level of reduction in AHL signal production as the BDSF-minus mutant ΔrpfF_Bc_ (Figure 3A). Given that binding of BDSF by RpfR could substantially increases its activity in c-di-GMP degradation [14], it is rational that increasing c-di-GMP level would lead to down-regulation of the AHL signal production and that decreasing c-di-GMP level would promote AHL signal production. Consisting with the above reasoning, our results showed that *in trans* expression of the c-di-GMP synthases, WspR from *P. aeruginosa* or the GGDEF domain of RpfR, in wild type H111 led to decreased AHL production (Figure 4), and that reducing c-di-GMP level in the BDSF-minus mutant ΔrpfF_Bc_ by overexpressing either RocR from *P. aeruginosa* or the EAL domain of RpfR resulted in increased AHL signal biosynthesis (Figure 4). These findings have elucidated a signaling pathway with which the BDSF-type QS system regulates the AHL-type QS system in *B. cenocepacia* and, additionally, have also further expanded our understanding of the c-di-GMP signaling mechanisms in modulation of bacterial physiology. However, how c-di-GMP controls AHL signal production remains to be further investigated.

**Figure 7:**
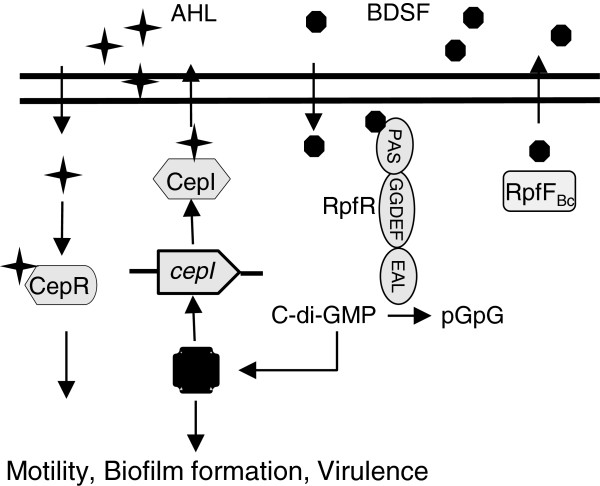
**Schematic representation of the QS signalling networks in *B. cenocepacia. ***RpfR_Bc_ and CepI are involved in synthesis of BDSF and AHL signals, respectively. Perception of BDSF by RpfR substantially enhances its c-di-GMP phosphodiesterases activity and causes a reduction of the intracellular c-di-GMP level, and consequently affects the *cepI* transcriptional expression level and a range of biological functions, including swarming motility, biofilm formation and virulence through an unknown c-di-GMP effector X. The AHL-dependent QS system is also implicated in regulation of motility, biofilm formation, and virulence through its cognate receptor CepR. Solid arrows indicate the signalling regulation or signal transport.

Identification of the second messenger c-di-GMP as a key element in the BDSF/c-di-GMP/AHL signaling pathway is also critical for explanation of the seeming puzzling relationship between BDSF and AHL systems in regulation of bacterial physiology and virulence and for elucidation of the QS regulatory mechanisms in *B. cenocepacia* H111. Our data showed that both BDSF and AHL systems control similar phenotypes including bacterial motility, biofilm formation and protease production with an obvious cumulative effect (Figure 5). How these two QS systems interact in regulation and coordination of various biological functions? Do they act in cascade or independently? Our data support a partial “cascade” and a partial “independent” signaling mechanisms. Firstly, knocking out BDSF production affects AHL production but only partially reduced the total AHL level (Figure 1). Secondly, null mutation of RpfR, which acts as a net c-di-GMP degradation enzyme upon interaction with BDSF [14], showed an almost identical effect on AHL signal production as the BDSF-minus mutant (Figure 3). Thirdly, double deletion of the BDSF synthase gene *rpfF*_*Bc*_ and the AHL synthase gene *cepI* showed a more severe impact on bacterial physiology and virulence than the corresponding single-deletion mutants (Figures 5 and 6). Finally, exogenous addition of either BDSF or AHL could only partially rescue the changed phenotypes of the double deletion mutant ΔrpfF_Bc_ΔcepI but a combination of BDSF and AHL could completely restore the changed phenotypes (Figure 5). These findings, together with the previous knowledge of the second messenger c-di-GMP, suggest a working model of QS network in *B. cenocepacia* H111 in which BDSF and AHL elements are linked through the second messenger c-di-GMP (Figure 7). Considering that c-di-GMP is widely associated with the regulation of various biological functions, including motility, biofilm formation and virulence factor production [10,24,25], it is highly likely that BDSF system could influence the downstream gene expression through modulating the intracellular levels of both c-di-GMP and AHL signals. On the other hand, the AHL system could also act independently in regulation of downstream genes in the absence or presence of BDSF as the AHL signal production is only partially controlled by the BDSF system. In summary, the findings presented in this study have outlined a novel and flexible multicomponent QS network, which consists of BDSF and AHL QS systems and the second messenger c-di-GMP, in *B. cenocepacia* H111. This regulatory network has an interesting feature that both BDSF and AHL systems could act either together or independently in modulation of bacterial physiology and virulence, which may offer competitive advantages and flexibility in pathogen-host and microbe-microbe interactions.

## Conclusions

The QS signal BDSF controls AHL signal production through regulation of the AHL synthase CepI expression at transcriptional level by modulating the intracellular level of the second messenger c-di-GMP through its novel receptor RpfR. The two QS systems have a cumulative role in regulation of various biological functions, including swarming motility, biofilm formation and virulence factor production. Exogenous addition of either BDSF or AHL signal molecules could only partially rescue the changed phenotypes of the double deletion mutant defective in BDSF and AHL signal production.

## Methods

### Bacterial growth conditions and virulence assays

Bacterial strains used in this work are listed in Table 1. *B. cenocepacia* strains were cultured at 37°C with shaking at 200 rpm in NYG medium (5 g peptone, 3 g yeast extract, and 20 g glycerol per liter) [33]. The following antibiotics were supplemented when necessary: tetracycline, 100 μg ml-1; ampicillin, 200 μg ml-1; trimethoprim, 25 μg ml-1. Killing assays were performed using *Caenorhabditis elegans* strain Bristol N2. Nematodes were maintained on nematode growth medium (NGM) at 23°C [34]. Slow killing assays were performed on NGM medium and fast killing assays on high-osmolarity PGS medium (peptone-glucose-sorbitol) [22]. BDSF and OHL signal molecules were added to the medium at a final concentration of 5 μM unless indicated otherwise.

**Table 1:** Bacterial strains and plasmids used in this study

**Strain or plasmid**	**Phenotypes and/or characteristics**	**Reference or source**
***B. cenocepacia***		
WT	Wild type strain H111, Genomovars III of the *B. cepacia* complex	23
WT(GGDEF)	Wild type strain harboring the expression construct pLAFR3-GGDEF	14
WT(wspR)	Wild type strain harboring the expression construct pMLS7-wspR	This study
∆rpfF_Bc_	BDSF-minus mutant derived from H111 with *rpfF*_*Bc*_ being deleted	14
∆rpfF_Bc_(EAL)	Mutant ∆rpfF_Bc_ harboring the expression construct pLAFR3-EAL	14
∆rpfF_Bc_(rocR)	Mutant ∆rpfF_Bc_ harboring the expression construct pMLS7-rocR	14
∆rpfF_Bc_(wspR)	Mutant ∆rpfF_Bc_ harboring the expression construct pMLS7-wspR	This study
∆rpfF_Bc_ (rpfF_Bc_)	Mutant ∆rpfF_Bc_ harboring the expression construct pMLS7-rpfFBc	14
∆rpfF_Bc_ (cepI)	Mutant ∆rpfF_Bc_ harboring the expression construct pMLS7-cepI	This study
∆rpfF_Bc_ (PcepI-lacZ)	Mutant ∆rpfF_Bc_ harboring the expression construct PcepI-lacZ	This study
∆rpfR	Deletion mutant with *rpfR* being deleted	14
∆rpfR(rpfR)	Mutant ∆rpfR harboring the expression construct pMLS7-rpfR	14
∆rpfR(rpfRAAL)	Mutant ∆rpfR harboring the expression construct pMLS7-rpfRAAL	This study
∆rpfR(rpfRGGAAF)	Mutant ∆rpfR harboring the expression construct pMLS7-rpfRGGAAF	This study
∆cepI	Deletion mutant with *cepI* being deleted	23
∆cepI(rpfF_Bc_)	Mutant ∆cepI harboring the expression construct pMLS7-rpfF_Bc_	This study
∆rpfF_Bc_∆cepI	Double deletion mutant with *rpfF*_*Bc*_ and *cepI* being deleted	This study
∆rpfR (PcepI-lacZ)	Mutant ∆rpfR harboring the expression construct PcepI-lacZ	This study
BCAM0227 (PcepI-lacZ)	Insertional mutant of BCAM0227 harboring the expression construct PcepI-lacZ	This study
*E. coli*		
DH5α	*supE44 ∆lacU169(Φ80lacZ∆M15) hsdR17 recA1 endA1 gyrA96 thi-1 relA1 λpir*	Laboratory collection
OP50	Food source of *C. elegans*	22, 34
*Agrobacterium tumefaciens*		
CF11	AHL reporter strain	Lab of Stephen K. Farrand
Plasmid		
pMLS7-rpfF_Bc_	pMLS7 containing *rpfF*_*Bc*_	12
pMLS7-rpfR	pMLS7 containing *rpfR*	14
pMLS7-rpfRAAL	pMLS7-rpfR harboring an E443A amino acid substitution	This study
pMLS7-rpfRGGAAF	pMLS7-rpfR harboring a D318A and E319A amino acid substitution	This study
pMLS7-cepI	pMLS7 containing *cepI*	13
pMLS7-wspR	pMLS7 containing *wspR*	This study
pMLS7-rocR	pMLS7 containing *rocR*	This study
pLAFR3-GGDEF	pLAFR3 containing the encoding region of the GGDEF domain of RpfR	14
pLAFR3-EAL	pLAFR3 containing the encoding region of the EAL domain of RpfR	14
PcepI-lacZ	pME2-lacZ containing promoter of *cepI*	This study

### Construction of in-frame deletion mutants and complementation strains

The *cepI* deletion mutant of *B. cenocepacia* strain H111 was used as the parental strain to generate the in-frame double deletion mutant of *rpfF*_*Bc*_ and *cepI*, following the methods described previously [12]. For complementation analysis, the coding region of WspR was amplified by PCR using the primers listed in Additional file 4: Table S1, and cloned under the control of the S7 ribosomal protein promoter in plasmid vector pMSL7. The resultant construct was conjugated into the *rpfF*_*Bc*_ deletion mutant *B. cenocepacia* H111 using tri-parental mating with pRK2013 as the mobilizing plasmid.

### Construction of reporter strains and measurement of β-galactosidase activity

The promoter of *cepI* was amplified using the primer pairs listed in Additional file 4: Table S1 with HindIII and XhoI restriction sites attached. The resulting products were digested with HindIII and XhoI, and ligated at the same enzyme sites in the vector pME2-lacZ [35]. These constructs, verified by DNA sequencing, were introduced into *B. cenocepacia* H111 using tri-parental mating with pRK2013. Transconjugants were then selected on LB agar plates supplemented with ampicillin and tetracycline. Bacterial cells were grown at 37°C and harvested at different time points as indicated, and measurement of β-galactosidase activities was performed following the methods as described previously [36].

### Biofilm formation, swarming motility and proteolytic activity assays

Biofilm formation in 96-well polypropylene microtiter dishes was assayed essentially as described previously [23]. Swarming motility was determined on semi-solid agar (0.5%). Bacteria were inoculated into the center of plates containing 0.8% tryptone, 0.5% glucose, and 0.5% agar. The plates were incubated at 37°C for 18 h before measurement of the colony diameters. Protease assay was performed following the previously described method [37]. Protease activity was obtained after normalization of absorbance against corresponding cell density.

### Analysis of AHL signals

Bacterial cells were grown in NYG medium to a same cell density in the late growth phase. The supernatants were acidified to pH = 4.0 and extracted using ethyl acetate in a 1:1 ratio. Following evaporation of ethyl acetate the residues were dissolved in methanol. Quantification of AHL signals was performed using β-galactosidase assay with the aid of the AHL reporter strain CF11 as described previously [38]. Briefly, the reporter strain was grown in minimal medium at 28°C with shaking at 220 rpm overnight. The cultures were inoculated in the same medium supplemented with extracts containing AHL signals. Bacterial cells were harvested and β-galactosidase activities were assayed as described in previous section. For TLC analysis, 5 μl of the concentrated AHL extracts were spotted onto 10 × 20 cm RP-18254 s plate (MERCK) and separated with methanol–water (60:40, v/v). The plates were subsequently air dried and overlaid with 50 ml minimal medium containing 0.8% agarose, 50 μg ml-1 X-gal, and 1 ml stocked CF11 culture. The plates were then incubated overnight at 28°C, and AHL is indicated by the presence of a blue spot.

### Western blotting analysis

Bacterial cultures were grown in NYG medium overnight and inoculated in the same medium. The refreshed cultures were grown at 37°C to an OD_600_ of 4.5; and 1 ml of each bacterial culture was collected and centrifuged. The cells were lysed by adding 250 μl celLyticTM B cell Lysis Reagent (Sigma). The concentrations of total protein samples were measured and normalized. Then the samples were denatured by boiling for 10 min and separated by 10% SDS-PAGE. Western blot analysis was performed following the standard protocols [39].

## Authors’ contributions

Experiments were carried out by YD, AL, JW, TZ, SC, JL, YHD. Data analysis was finished by YD and LHZ. The study was designed by YD and LHZ, who also drafted the manuscript. All authors read and approved the final manuscript.

## Supplementary Material

Additional file 1: Figure S1 Mutation of *BCAM0227* does not affect *cepI* expression level. Click here for file

Additional file 2: Figure S2 Complementation of *rpfR* with RpfR, RpfR_AAL_ and RpfR_GGAAF_. Click here for file

Additional file 3: Figure S3 Cumulative effect of BDSF and AHL systems in regulation of bacterial motility, biofilm formation, and protease production. Click here for file

Additional file 4: Table S1 Primers used in this study. Click here for file
