# Supplementary material for: Cis-2-dodecenoic acid quorum sensing system modulates N-acyl homoserine lactone production through RpfR and cyclic di-GMP turnover in Burkholderia cenocepacia
Source: BMC Microbiol. 2013 Jul 1;13:148. doi: 10.1186/1471-2180-13-148 (PMC3703271; doi:10.1186/1471-2180-13-148)
Supplement: Additional file 1: Figure S1 — Mutation of BCAM0227 does not affect cepI expression level. [file 1471-2180-13-148-S1.doc]

**Fig. S1.** Mutation of *BCAM0227* does not affect *cepI* expression level. The β-galactosidase activity of a *cepI*-*lacZ* transcriptional fusion was tested in wild-type, △rpfF_Bc_ and BCAM0227. For convenient comparison, the activity value in wild-type strain was defined as 100% and used to normalize the β-galactosidase activity of the different mutant strains. The data presented are the means of three replicates and error bars represents the standard deviation.
