# Supplementary material for: Cis-2-dodecenoic acid quorum sensing system modulates N-acyl homoserine lactone production through RpfR and cyclic di-GMP turnover in Burkholderia cenocepacia
Source: BMC Microbiol. 2013 Jul 1;13:148. doi: 10.1186/1471-2180-13-148 (PMC3703271; doi:10.1186/1471-2180-13-148)
Supplement: Additional file 2: Figure S2 — Complementation of rpfR with RpfR, RpfRAAL and RpfRGGAAF. [file 1471-2180-13-148-S2.doc]

**Fig. S2**. Complementation of *rpfR* with RpfR, RpfR_AAL_ and RpfR_GGAAF_. *In trans* expression of RpfR and RpfR_GGAAF_ complemented AHL signal production of a RpfR deficient mutant, whereas *e*xpression of RpfR_AAL_ failed to restore the AHL signal production defect of a *rpfR* mutant background. For convenient comparison, the AHL signal production value of wild-type strain was defined as 100% and used to normalize the AHL signal production of other strains. The data shown are the means of three replicates and error bars indicate standard errors.
