# Supplementary material for: Cis-2-dodecenoic acid quorum sensing system modulates N-acyl homoserine lactone production through RpfR and cyclic di-GMP turnover in Burkholderia cenocepacia
Source: BMC Microbiol. 2013 Jul 1;13:148. doi: 10.1186/1471-2180-13-148 (PMC3703271; doi:10.1186/1471-2180-13-148)
Supplement: Additional file 3: Figure S3 — Cumulative effect of BDSF and AHL systems in regulation of bacterial motility, biofilm formation, and protease production. [file 1471-2180-13-148-S3.doc]

**Fig. S3.** Cumulative effect of BDSF and AHL systems in regulation of bacterial motility (A), biofilm formation (B), and protease production (C). The data presented are the means of three replicates and error bars represents the standard deviation.

A
