# Supplementary material for: Cis-2-dodecenoic acid quorum sensing system modulates N-acyl homoserine lactone production through RpfR and cyclic di-GMP turnover in Burkholderia cenocepacia
Source: BMC Microbiol. 2013 Jul 1;13:148. doi: 10.1186/1471-2180-13-148 (PMC3703271; doi:10.1186/1471-2180-13-148)
Supplement: Additional file 4: Table S1 — Primers used in this study. [file 1471-2180-13-148-S4.doc]

**Table S1.** Primers used in this study

| Primer | Sequence (5’-3’) |
| --- | --- |
| wspR-F | TGCTCTAGAATGCACAACCCTCATGAGAGCAAG |
| wspR-R | CCCAAGCTTCTGCACTTGCGCCCCACAGG |
| cepI-pro-F | CCCAAGCTTGGGCGTTCGAGGCTGATACATTCCG |
| cepI-pro-R | CCGCTCGAGCGGCCGCCCTTCCTCGTGAACGAA |
| AAL-F | GGGCGACGTGCACGGCGTCGCGGCGCTGATCC |
| AAL-R | GGATCAGCGCCGCGACGCCGTGCACGTCGCCC |
| GGAAF-F | GCGCGGCTCGGCGGCGCCGCATTCCTCGTGCTGTT |
| GGAAF-R | AACAGCACGAGGAATGCGGCGCCGCCGAGCCGCGC |
|  |  |
|  |  |
